# Supplementary material for: Adaptive Laboratory Evolution of a Microbial Consortium Enhancing Non-Protein Nitrogen Assimilation for Feed Protein Production
Source: Microorganisms. 2025 Jun 18;13(6):1416. doi: 10.3390/microorganisms13061416 (PMC12195778; doi:10.3390/microorganisms13061416)
Supplement: Supplementary file 1 [file microorganisms-13-01416-s001.zip › microorganisms-3669803-supplementary.pdf]

Supplementary Materials

Adaptive laboratory evolution of a microbial consortium enhancing non-protein nitrogen assimilation for feed protein production

Yi He [1,†], Shilei Wang [1,4,†], Yifan Mi [1], Mengyu Liu [1], Huimin Ren [1], Zhengxiang Guo [1], Zhen Chen [2], Yafan Cai [1,4], Jingliang Xu [1,4], Dong Liu [3], Chenjie Zhu [3], Zhi Wang [1,4,*], Hanjie Ying [3]

[1] School of Chemical Engineering, Zhengzhou University, Zhengzhou 450001, Henan, China

[2] Henan Key Laboratory of Tea Plant Biology, College of Life Science, Xinyang Normal University, Xinyang, China

[3] National Engineering Research Center for Biotechnology, Nanjing Tech University, Nanjing 211816, Jiangsu, China

[4] State Key Laboratory of Biobased Transport Fuel Technology, Zhengzhou University, Zhengzhou 450001, China

[†] These authors contribute equally to this work.

[*] Correspondence: zhiwang@zzu.edu.cn

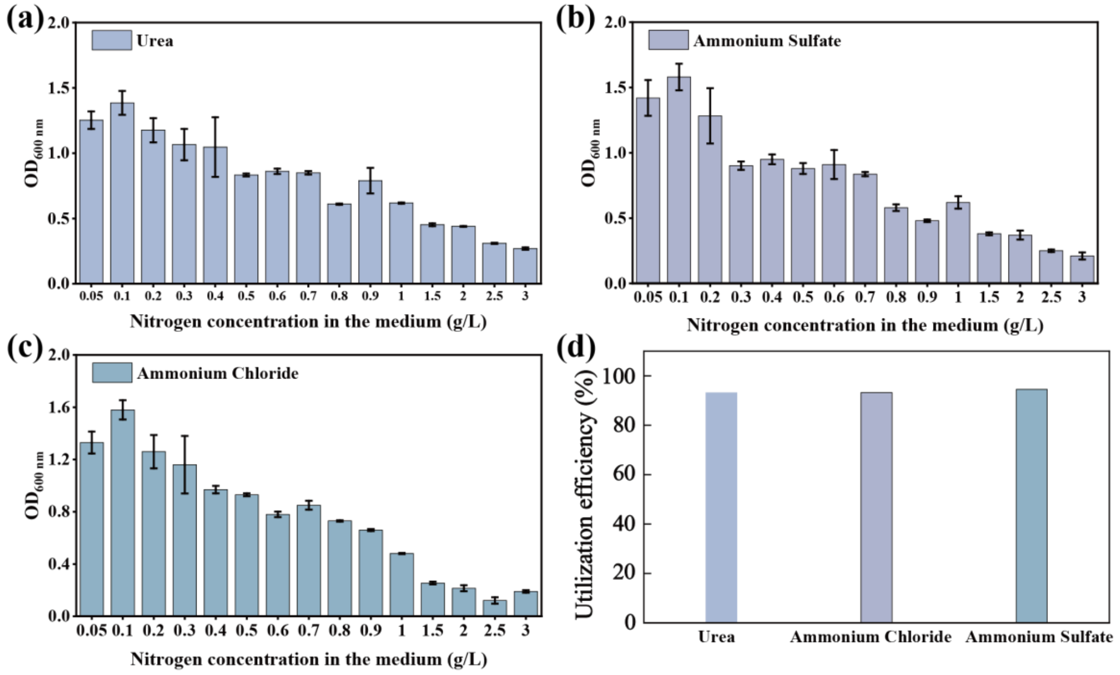

Figure S1 The growth of the original microbial consortium in medium with different concentrations of urea (a), ammonium chloride (b), and ammonium sulfate (c) as the sole nitrogen source and wheat straw as the sole carbon source. The data represent the averages of biological triplicates, and the scale bar represents the standard deviation (SD).

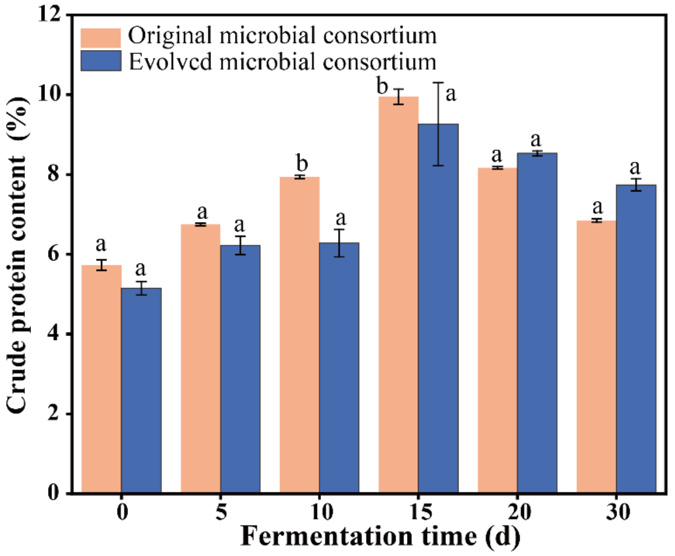

Figure S2 The crude protein of the wheat straw fermented by using the original and the evolved microbial consortium with the addition of ammonium sulfate as nitrogen source. The data represent the averages of biological triplicates, and the scale bar represents the standard deviation (SD), different lowercase letters indicate significant differences between treatments ($P < 0.05$).

Table S1 The concentrations of NPN at different generations during the ALE.

generations of domestication	Non-Protein Nitrogen (NPN) Concentration (g/L)	Urea Concentration (g/L)	Ammonium Chloride Concentration (g/L)	Ammonium Sulfate Concentration (g/L)
0~5	0.1	0.214	0.382	0.472
6~8	0.2	0.429	0.764	0.944
9~15	1.0	2.143	3.821	4.719
16~20	2.0	4.286	7.641	9.439

Table S2 Changes of protein content and NPN content during the scale-up fermentation process

Fermentation time (d)	True protein content (%)	Crude protein content (%)	NPN content (%)
0	2.74	8.04	0.91
20	11.60	15.34	0.15
